# Laboratory evaluation of immunochromatographic rapid diagnostic tests for cholera in Haiti

**DOI:** 10.1371/journal.pone.0186710

**Published:** 2017-11-01

**Authors:** Wilfredo R. Matias, Fabrice E. Julceus, Cademil Abelard, Leslie M. Mayo-Smith, Molly F. Franke, Jason B. Harris, Louise C. Ivers

**Affiliations:** 1 Department of Global Health & Social Medicine, Harvard Medical School, Boston, Massachusetts, United States of America; 2 Partners In Health, Boston, Massachusetts, United States of America; 3 Zanmi Lasante, Pont-Tambour, Saint Marc, Haïti; 4 Division of Infectious Diseases, Massachusetts General Hospital, Boston, Massachusetts, United States of America; 5 Department of Pediatrics, Harvard Medical School, Boston, Massachusetts, United States of America; 6 Division of Global Health Equity, Brigham & Women’s Hospital, Boston, Massachusetts, United States of America; Johns Hopkins Bloomberg School of Public Health, UNITED STATES

## Abstract

**Background:**

Rapid diagnostic tests (RDT) for cholera are promising tools for detecting cholera in areas with limited laboratory infrastructure. However, evidence on the characteristics of the many available RDTs is scarce, and their use has been limited by suboptimal performance. We evaluated the performance characteristics of three cholera RDTs from Span Diagnostics, Artron Laboratories, and Standard Diagnostics in a regional laboratory in Haiti.

**Methodology/Principal findings:**

We retrospectively reviewed records from May 2014 to October 2015 of a laboratory-based surveillance program for *Vibrio cholerae* at Hôpital Saint-Nicolas in Saint-Marc, Haiti. We compared the results of 511 Crystal VC, 129 Artron and 451 SD Bioline RDTs to bacterial culture as the gold standard. Of 905 cultures, 477 (52.7%) were positive for *V*. *cholerae* O1, of which 27.7% were serotype Inaba. No cultures grew *V*. *cholerae* O139. Sensitivity and specificity of Crystal VC were 98.6% (95%CI: 96.5%-99.6%) and 71.1% (95%CI: 64.7%-76.9%), respectively. Artron demonstrated a sensitivity of 98.6% (95%CI: 92.7%-100%) and specificity of 69.1% (95%CI: 55.2%-80.9%). SD Bioline demonstrated a sensitivity of 81.1% (95%CI: 75.6%-85.8%) and specificity of 92.8% (95%CI: 88.4%-95.9%). Crystal VC and Artron frequently showed false positive O139 bands, whereas none were seen with SD Bioline.

**Conclusions/Significance:**

There is significant variation in the performance of different cholera diagnostic RDTs. Artron and Crystal VC RDTs have high sensitivity and low specificity, while SD Bioline RDT has low to moderate sensitivity and high specificity when performed by laboratory technicians in Haiti. Study limitations included its retrospective design. The suboptimal characteristics of these tests limit their use as clinical point-of-care tests; however, they may be useful in outbreak response, surveillance, and research in resource-limited settings.

## Introduction

Cholera remains a significant cause of morbidity and mortality worldwide, resulting in an estimated 2.8 million cases and 91,000 deaths annually.[1] Epidemics such as that in Haiti have highlighted the debilitating impact of cholera on underserved regions already lacking the robust water, sanitation, and healthcare systems required to prevent and control outbreaks.[2] However, recent years have also brought significant advances in our understanding of the clinical and public health approaches to combat the disease.[3] The mobilization of these cholera control strategies ultimately depends on the timely and accurate identification of cholera cases.

Prompt identification of cholera cases facilitates rapid outbreak responses in the short term, while providing reliable surveillance data to guide long-term policies and interventions. To this end, microbiological stool culture, the current recognized gold standard for the diagnosis of cholera has significant limitations.[4] Culture to isolate and identify the causative bacterium, *Vibrio cholerae*, may take up to 3 days to complete and requires laboratory capacity that is often absent in underserved settings.[5] Furthermore, the accuracy of culture methods and their reliability as gold standards are increasingly being called into question due to their suboptimal sensitivity.[3,5,6] PCR-based technologies, although more accurate than stool culture, are rarely available in settings most afflicted by cholera.[5]

Rapid diagnostic tests (RDTs) represent promising alternatives for the diagnosis of cholera in underserved settings.[7] Current cholera RDTs are lateral flow devices that detect the lipopolysaccharide of *V*. *cholerae* O1 and O139 via immmunochromatographic assays.[5] They are fast, affordable, easy to use, and require minimal infrastructure. As such, they are well suited to meet the demand for cholera diagnosis in areas where culture or PCR based methods are not feasible. Despite their advantages, there are also significant limitations to current RDTs; evidence to guide their use is limited, and most studies conducted with Crystal VC, the most widely used RDT, demonstrate suboptimal specificity.[8] For example, previous evaluations of Crystal VC have demonstrated a sensitivity ranging from 58%–100% and specificity ranging from 60%–100%.[6–17] In Haiti, this test has been evaluated twice, demonstrating sensitivities of 95 and 95.6%, and specificities of 79.5% and 80%.[12,18] A recent technical note by the Global Task Force for Cholera Control of the World Health Organization highlights these limitations in performance and recommends limiting the use of these tests to surveillance purposes, while providing guidance for further development and implementation of RDTs.[7]

To address some of these knowledge gaps, we evaluated the performance characteristics of three commercially available immunochromatographic RDTs. These included the Crystal VC Dipstick (Arkray Healthcare Pvt., India; previously Span Diagnostics, Surat, India), SD Bioline Cholera Ag O1/O139 RDT (Standard Diagnostics Inc., Korea) and Artron *Vibrio cholerae* O139 and O1 Combo Test (Artron Laboratories Inc., Canada). Information on the different characteristics of the 3 RDTs as reported on the manufacturers’ package insert are found in Table 1.

**Table 1:** Descriptive characteristics of Crystal VC, Artron and SD Bioline RDTs for cholera.

Test Name	Manufacturer	Intended Use (per company)	Reported Sensitivity	Reported Specificity
Crystal VC Dipstick	Arkray Healtchare Pvt., India *	Rapid visual immunochromatographic test for qualitative and differential detection of *V*. *cholerae* O1 and O139 in stool.	88–100%	61–87.3%
Artron *Vibrio cholerae* O139 and O1 Combo Test	Artron Laboratories Inc., Canada	Rapid, convenient immunochromatographic assay for the qualitative detection of either *Vibrio cholerae* O139 or O1 in human fecal samples or environmental water.	99%	99%
SD Bioline Cholera Ag O1/O139 RDT	Standard Diagnostics Inc., South Korea	Rapid, qualitative test for the detection of *V*. *cholerae* O1 and O139 antigens in human fecal specimens.	95.4%	94.1%

*At the time study was performed this test was manufactured by Span Diagnostics Ltd., India.

## Materials and methods

### Study setting and population

This study was conducted at Hôpital Saint Nicolas in Saint Marc, Artibonite, Haiti. Cholera is now endemic in Haiti after having been introduced in 2010.[19] The study site is a 200-bed inpatient referral hospital run by the Ministry of Health (MSPP) with the support of the international organization, Partners In Health. The hospital runs a cholera treatment center (CTC) that has actively treated cases of acute watery diarrhea since the onset of the cholera epidemic in 2010. It recently established an Enteric Diseases Laboratory in 2014 providing diagnostic capacity for cholera and other diarrheal diseases for the region. The CTC serves a catchment area of approximately 1 million individuals including the surrounding urban population of Saint Marc, rice farmers living along the rural banks of the Artibonite River, and individuals travelling from surrounding areas seeking healthcare.

### Study design

This study was a retrospective review of diarrheal disease surveillance records in the Enteric Diseases Laboratory at the study site in Haiti between May 1^st^, 2014 and October 15^th^, 2015. All specimens that underwent both RDT testing and culture for identification of *V*. *cholerae* and had complete records were included in our data collection and analysis.

### Specimen collection and bacterial culture

As part of hospital-based surveillance activities, stool specimens were collected from cases presenting to the CTC meeting criteria for acute watery diarrhea (defined as 3 or more non-bloody bowel movements in a 24h period, with symptoms lasting less than 7 days). All cases meeting the definition for acute watery diarrhea (i.e. cholera suspect cases) were provided with specimen collection cups and instructed how to appropriately collect a sample by health care providers in the CTC. Specimens were then transferred to the Enteric Diseases Laboratory and processed by trained laboratory technicians. Specimens underwent rapid diagnostic testing immediately upon arrival. For bacterial culture, specimens were either processed immediately upon arrival, or stored in Cary Blair (Becton, Dickinson and Co., Maryland, USA) transport medium at 2-8C and processed within a week of arrival. Selective thiosulfate citrate bile salts sucrose agar (TCBS) (Becton, Dickinson and Co., Maryland, USA) plates were inoculated with either an inoculum from liquid stool or a Cary Blair swab and incubated for 18 to 24 hours at 35° to 37°C. Between May 1^st^, 2014 and May 22^nd^, 2015, specimens were plated directly onto TCBS without prior enrichment in alkaline peptone water (APW). APW became available at the study site on May 22^nd^, 2015; to improve their yield, all cultures following that date were enriched in APW for 6 hours prior to plating on TCBS.[20] Candidate colonies following overnight growth were sub-cultured on heart infusion agar (HIA) (Becton, Dickinson and Co., Maryland, USA), a non-inhibitory medium. The HIA plate was then incubated at 35° to 37°C for up to 24 hours. Overnight growth from HIA plates were examined by Gram staining. Isolates from HIA underwent biochemical testing via the string and oxidase tests, and definitive identification via serologic slide agglutination with polyvalent O1 (Becton, Dickinson and Co., Maryland, USA) and O139 (Denka Seiken Co Ltd., Tokyo, Japan) antiserum and if positive for O1, monovalent Ogawa (Becton, Dickinson and Co., Maryland, USA) and Inaba (Becton, Dickinson and Co., Maryland, USA) antisera.

### Rapid diagnostic testing

RDTs were performed immediately upon specimen arrival to the laboratory. Three different RDTs were performed, depending on availability, such that some specimens underwent testing with one, two, three, or none of the RDTs. Test availability varied throughout the study period. Tests were performed following the manufacturer’s instructions for each assay. In brief, for Crystal VC RDT, two drops of liquid stool sample were dispensed into a sample processing vial with extraction buffer. The sample and buffer were mixed by shaking. Four drops of the processed sample with buffer mixture were dispensed into a test tube, and the RDT dipstick was vertically inserted into the test tube and allowed to sit for 15–20 minutes. For the Artron RDT, 6 drops of liquid stool sample were dispensed into a sample processing vial with extraction buffer and mixed. The test cassette was placed on a flat surface, and 3 drops of the specimen mixture were dispensed directly onto the sample well of the test cassette. Results were interpreted within 15 minutes. For the SD Bioline RDT, an assay diluent was transferred to a sample collection tube. A sample collection swab was inserted into the stool specimen, and then transferred into the diluent containing sample collection tube. The swab was vigorously rotated to mix the specimen with the solution. A dropping cap was then placed on the sample collection tube, and then used to dispense 4 drops into the sample well of the cassette. All three tests had a control band, and two bands specific for *V*. *cholerae* O1 and O139. Absence of a pink or red band at the control region indicated an invalid result. Presence of a pink or red band at the control region with the presence of a band at the O1 and O139 region indicated positivity for O1 and O139, respectively.

### Data management and statistical analysis

Bacteriological culture was used as the reference standard for comparison. Sensitivity, specificity, positive and negative predictive values for all three RDTs were calculated. Uncertainty was quantified by 95% confidence intervals using the exact method. We used Fisher’s exact test to compare the sensitivity and specificity of the RDTs depending on whether cultures were performed with or without APW enrichment. Performance characteristics for each RDT were evaluated separately; head-to-head comparisons were not performed. Calculations were done using STATA Version 14 (StataCorp, LP, College Station, TX, USA).

### Ethics statement

Study protocols were submitted and exempted from IRB review by the Harvard Medical School IRB Committee and the IRB Committee of Zanmi Lasante.

## Results

**Fig 1:**
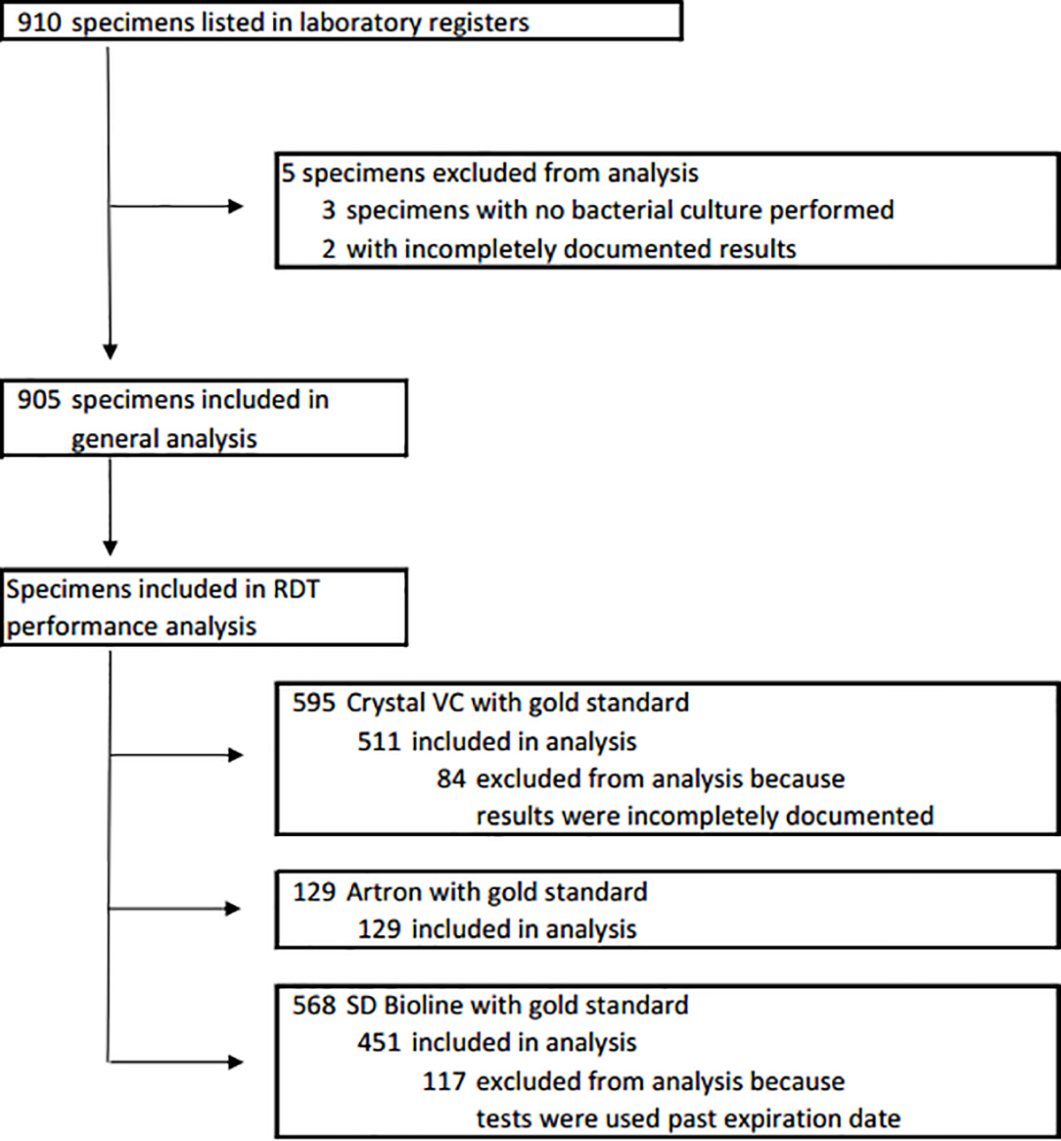
Profile of study specimens.

Between the period of May 1^st^, 2014 and October 15^th^, 2015, 910 specimens from patients presenting for acute watery diarrhea at the study site underwent testing for *V*. *cholerae*. (Fig 1) Of the 910 documented specimens, 905 had a documented result for bacterial culture and were included in the analysis. 3 specimens had no documented bacterial culture performed and 2 had incompletely documented results and so were excluded from the analysis. (Fig 1) 455 bacterial cultures were performed with APW enrichment and 450 without APW. Of all cultures performed, 477 (52.7%) were positive for *V*. *cholerae* O1, of which 345 (72.3%) were serotype Ogawa and 132 (27.7%) were serotype Inaba. 428 (47.3%) samples were negative for *V*. *cholerae* O1 or O139. Of these 428 negative specimens, 41 were isolated on TCBS, grew on HIA and were string test and oxidase test positive, but were negative on serologic testing with antisera for *V*. *cholerae* O1 and O139. No samples were culture positive for *V*. *cholerae* O139. For a breakdown of the number of cultures and RDTs performed with or without APW enrichment, see S1 Table. 596 Crystal VC RDTs were performed; 511 were included in the analysis, and 84 were excluded because results were incompletely documented.129 Artron RDTs were performed and included in the analysis. 568 SD Bioline RDTs were performed; 451 were included in the analysis, and 117 were excluded because these tests were performed past their expiration date. (Fig 1) RDT test results for the detection of *V*. *cholerae* O1 and O139 are listed in Table 2. 349 of the 511 Crystal VC RDTs and 428 fo the 451 SD Bioline RDTs, and 9 of the 129 Artron RDTs were compared to bacterial cultures that did not go APW enrichment. (S1 Table) For a list of the number of specimens tested by different combinations of RDTs see S2 Table.

**Table 2:** Results of 3 RDTs for the detection of *V*. *cholerae* O1 and O139.

		Culture		
		Positive	Negative	Total
Crystal VC	O1	189	29	218
	O1O139	93	36	129
	O139	1	25	26
	Negative	3	135	138
	Total	286	225	511
Artron	O1	70	11	81
	O1O139	3	6	9
	O139	0	3	3
	Negative	1	35	36
	Total	74	55	129
SD Bioline	O1	197	15	212
	O1O139	0	0	0
	O139	0	0	0
	Negative	46	193	239
	Total	243	208	451

The sensitivity and specificity for the detection of *V*. *cholerae* O1 of the 3 RDTs evaluated in this study, without consideration of O139 positive RDT results, were as follows. The sensitivity and specificity of Crystal VC was 98.6% and 71.1%, respectively. Similarly, Artron RDT demonstrated a sensitivity of 98.6% and specificity of 69.1%. The Sensitivity of SD Bioline was 81.1%, and its specificity was 92.8%. Overall sensitivity, specificity, positive predictive value and negative predictive value with accompanying 95% confidence intervals for the 3 RDTs evaluated are shown in Table 3. Comparison of performance characteristics depending on whether corresponding bacterial cultures were performed with or without prior APW enrichment showed no significant differences except for differences in the sensitivity of SD Bioline RDT. The sensitivity of SD Bioline was 57.1% (95%CI 28.9%– 82.3%, N = 23) with APW and 82.5% (95%CI 77.0%– 87.2%, N = 428) without APW (Two-sided Fisher’s exact test, P = 0.03).

**Table 3:** Performance characteristics of 3 RDTs compared to bacterial culture as the gold standard for the detection of *V*. *cholerae* O1.

	Crystal VC		Artron		SD Bioline	
	%	95% CI	%	95% CI	%	95% CI
Sensitivity	98.6	96.5–99.6	98.6	92.7–100.00	81.1	75.6–85.8
Specificity	71.1	64.7–76.9	69.1	55.2–80.9	92.8	88.4–95.9
Positive Predictive Value	81.3	76.8–85.2	81.1	71.5–88.6	92.9	88.6–96.0
Negative Predictive Value	97.6	93.9–99.3	97.4	86.5–99.9	80.8	75.2–85.6

Performance characteristics for the detection of *V*. *cholerae* O1 were also calculated assuming that either an O1 or O139 positive band on the RDT was interpreted as a positive result. In this analysis, the sensitivity of Crystal VC was 99% and the specificity was 60%. The sensitivity of Artron RDT was 98.6% and the specificity was 63.6%. Sensitivity and specificity for SD Bioline RDT remained the same, no positive O139 bands were documented. Confidence intervals, positive predictive values and negative predictive values are listed in Table 4. We also calculated the specificity of Crystal VC, Artron and SD Bioline RDTs for the detection of *V*. *cholerae* O139. Crystal VC and Artron demonstrated a specificity of 69.7% (95%CI 65.4%–73.6%) and 90.7% (95%CI 85.0%–94.9%), respectively. SD Bioline demonstrated a specificity of 100% (95%CI 98.9%–100%).

**Table 4:** Performance characteristics of Crystal VC and Artron RDTs for the detection of *V*. *cholerae* O1 when O1 or O139 bands are considered positive.

	Crystal VC		Artron	
	%	95% CI	%	95% CI
Sensitivity	99.0	97.0–99.8	98.6	92.7–100.00
Specificity	60.0	53.3–66.5	63.6	49.6–76.2
Positive Predictive Value	75.9	71.2–80.1	78.5	68.8–86.3
Negative Predictive Value	97.8	93.8–99.5	97.2	85.5–99.9

**Fig 2:**
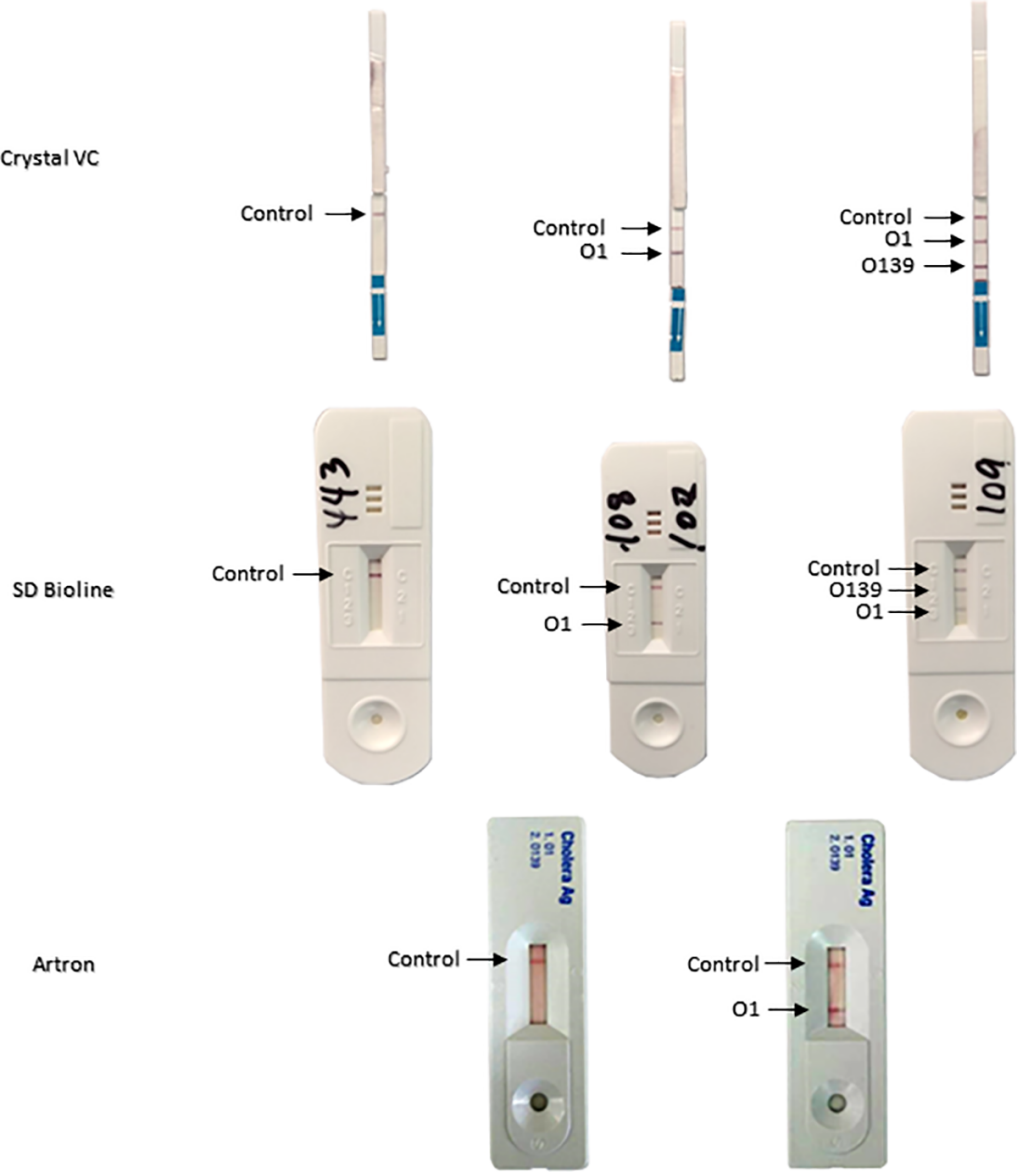
Representative images of positive Crystal VC, Artron and SD Bioline RDTs.

Of all the Crystal VC RDTs performed, 129 showed positive bands for O1 and O139 simultaneously. Of these, 93 were positive and 36 were negative for *V*. *cholerae* O1 by culture. 26 showed positive bands for O139 only, of which only one was positive for *V*. *cholerae* O1 by culture. Similarly, of all the positive Artron RDTs, 9 showed positive bands for O1 and O139 simultaneously, of which 3 were positive for *V*. *cholerae* O1 by culture. 3 showed a positive band for O139 only, of which all were negative for *V*. *cholerae* O1 by culture. No positive O139 bands were seen with all 451 SD Bioline RDTs performed either alone or in combination with a positive O1 bands. These data are summarized in Table 2. Representative photographs of positive Crystal VC RDT and Artron RDT bands are showed in Fig 2.

## Discussion

To our knowledge, this study represents the first published evaluation of Artron and SD Bioline RDTs for the diagnosis of infection with *V*. *cholerae* O1 compared to bacterial culture as the gold standard. Our study also adds to the growing evidence on the characteristics and utility of Crystal VC RDT. These results should help guide the future clinical, epidemiological and research use of these tests as part of global cholera prevention and control efforts.

The field of cholera diagnostics has lacked an accurate, rapid, affordable, and easy-to-use test that is optimized for diagnosis in the underserved settings most affected by cholera. Immunochromatographic RDTs, which seem most promising for this purpose, have been limited with regards to their availability, performance characteristics, and supporting evidence. A systematic review of diagnostic tests for cholera in 2012 demonstrated a notable absence of evidence to support the use of these rapid tests.[8] This review highlighted 5 RDTs that were particularly promising, and recommended further implementation and evaluation of these tests.[8] These tests included the Institut Pasteur (IP) dipstick, a prototype of the IP cholera dipstick that was commercialized as Crystal VC, the cholera SMART II test and the Cholera/Bengal Screen co-agglutination test (COAT), both by New Horizons Diagnostics, USA, and the Medicos dipstick by Advanced Diagnostics Inc., USA. Artron RDT and SD Bioline RDT were not among the 5 RDTs recommended by this review. Of these five recommended RDTs only Crystal VC (IP dipstick) and the SMART II test are currently commercialized. The availability of Artron RD and SD Bioline RDTs are welcome additions to currently available tools for diagnosing cholera. The sensitivity of Artron RDT in this study (98.6%) was similar to the sensitivity of Crystal VC measured in this study (98.6%) and others.[10,12–14,18] This test was recently developed and is now marketed by Artron Laboratories Inc., Canada.[21] It replaced Crystal VC as the diagnostic test distributed by the Haitian Ministry of Health in 2015. Its high sensitivity and similar performance characteristics make it an adequate alternative for Crystal VC. Although there have likely been manufacturer evaluations and small field evaluations, data regarding performance characteristics of SD Bioline RDT are not available in the published literature. In our study, although no direct comparisons were made, its sensitivity of 81.1% was lower than that of Crystal VC and Artron RDTs, and lower than the 95.4% sensitivity reported by the manufacturer. Its poor sensitivity limits its use as an effective cholera diagnostic test given the implications of a false negative diagnosis of a disease with such high epidemic potential.

Our findings also highlight the poor to modest specificity (71%) that has been a limitation of the current Crystal VC RDT. In this study, Artron RDT had a similarly low specificity of 69%. Interestingly, SD Bioline RDT had a specificity (93%) that was much higher than Crystal VC and Artron RDTs. It is possible that unique characteristics of the antibody used in this test increase its specificity at the expense of sensitivity. Efforts have been made to improve the specificity of Crystal VC RDT. Most evaluations were performed on an older version of the test, where the test was performed directly on stool. The dipstick has since been modified by the manufacturer to include a sample diluent buffer to which stool was added; this version of the test was used in this study. Our findings that the measured specificity of Crystal VC remains poor and similar to prior studies suggests that the use of this buffer may not improve the specificity. Reassuringly, it seems to not have affected the sensitivity of the test. Recent efforts by research groups have explored modifications of the Crystal VC RDT to address the low specificity that has been its major limitation. For example, the development of a modified protocol involving enrichment of stool samples in APW for 6 hours prior to use of the dipstick has significantly improved the specificity of the test.[22,23] This significantly increases the time required to perform the test from 20 minutes to over 6 hours, complicating its use in field settings; however, this is still faster than the two to three days required for bacterial culture, and its performance characteristics in a recent study were similar to culture, which may make them a better alternative for surveillance and research.[24] Pre-test enrichment with APW and other modified methods could also be used with Artron and SD Bioline RDTs.

Overall, the performance characteristics of all three RDTs evaluated in this study do not meet the expected minimal performance of a sensitivity of at least 90% and a specificity of at least 85% recommended by the Global Task Force for Cholera Control.[7] The suboptimal performance characteristics of Crystal VC, Artron and SD Bioline RDTs have significant implications for the current use of RDTs as part of cholera control strategies. If used as clinical point-of-care tests, results of the test would have little impact on immediate clinical management. It’s utility as a clinical screening tool would depend on the prevalence of cholera in the region. For example, in regions where cholera prevalence is low, the positive predictive value of the test would decrease, resulting in many false positives. Alternatively, in regions were prevalence is high, the test could be integrated into surveillance protocols, and used to identify specimens for culture or PCR confirmation in reference laboratories. This would expedite surveillance protocols while diminishing material costs. In their current form, these tests should not be used for the diagnosis of cholera in individual patients. Despite this, in Haiti and elsewhere, they are commonly use in clinical settings, to guide triage and treatment, and often affect the care of individual patients. When used as described by the manufacturer, these high-sensitivity, low-specificity tests such as Crystal VC and Artron should be reserved for epidemiologic and research purposes. For example, as is recommended by the Global Task Force for Cholera Control, samples from outbreaks of acute watery diarrhea of unknown etiology would be tested by RDTs, and if a number of samples above a threshold are positive, this should trigger a cholera alert and initiate response activities.[5,7]

In this study, we also frequently noted O139 positive bands with Crystal VC and Artron RDT, whereas none were noted with SD Bioline. *V*. *cholerae* O139 has been notably absent in Haiti with the exception of one non-toxigenic O139 isolate that was identified by the Haitian National Public Health Laboratories and the Center for Disease Control between April 2012 and March 2013.[18] No *V*. *cholerae* O139 were identified by culture in this study, suggesting that all the O139 positive bands in this study detected with Crystal VC and Artron RDTs were false positives. No O139 positive bands were noted with SD Bioline RDT. This finding has been documented in two prior studies where interestingly, samples that were O139 positive for *V*. *cholerae* by RDT lost positivity when 6-hours of APW enrichment was performed prior to RDT testing.[22,24] The reasons for this finding are unclear. Previous studies have demonstrated that *V*. *cholerae* serogroups O22 and O144, *Vibrio mimicus* and *Aeromonas trota* share antigens with *V*. *cholerae* O139.[25,26] Although this cannot be determined from this data, it is possible that these O139 false positive RDT results are detecting the presence of non-O1/non-O139 *V*. *cholerae*, other *Vibrio* species or other diarrheagenic bacteria such as *Aeromonas*. For example, 41 specimens in this study were isolated on TCBS, grew on HIA, and were string test and oxidase test positive, but were negative on serologic testing with *V*. *cholerae* O1 and O139 antisera. Other environmental factors may also be playing a role. Care should be taken when training users of the test, ensuring to highlight the appropriate interpretation of a positive O139 band, as this can affect overall interpretation of the test. Additionally, single serogroup tests are available and could be used in regions where only one serogroup is prevalent.

This study has some limitations. First, the retrospective nature of this study is a limitation. Because of its retrospective design, we were unable to conduct parallel testing that would allow head-to-head comparisons of the tests, or collect demographic and clinical information on the population that could have enabled informative subgroup analyses. As such, we were unable to account for potential confounders such as antigenic drift, antibiotic consumption, and seasonality. We were also unable to fully ensure complete blinding of technicians when performing the gold standard and the RDT, or ensure equal and systematic preservation in Cary Blair media. Several laboratory protocols were already in place to minimize these sources of error. For example, technicians were routinely trained in appropriate data collection and interpretation to minimize bias, and although blinding was not complete, the flow of specimens through the laboratory ensured that technicians performing cultures were usually blinded to the RDT result. Preservation on Cary Blair media prior to bacterial culture has often been performed in prior evaluations of cholera RDTs; all efforts were made to minimize the time of preservation on Cary Blair media.[10,11,18] Second, bacteriological culture was considered the gold standard at the time this study was performed, but the accuracy of culture methods and their reliability as gold standards are more frequently being called into question. The sensitivity of bacterial culture is likely suboptimal because of factors such as prior antibiotic use or possibly intrahost phage predation.[4] A previous study evaluating Crystal VC in the absence of a gold standard using Bayesian latent class modeling showed that the specificity of this test is likely higher than previous measures relying on culture as the gold standard.[6] In another study where samples were tested for *V*. *cholerae* by RDT and culture, half of the RDT positive, culture negative samples tested positive by multiplex PCR, suggesting that some of these RDT positives were in fact true positive results.[4] Artron RDT and SD Bioline RDT could be included in similar studies where they are compared to each other and other diagnostic methods to further clarify their characteristics. Furthermore, not all the cultures in this study were performed with APW-enriched samples. Previous studies support the utility of APW enrichment prior to plating on TCBS in increasing the sensitivity of culture.[20] Differences in the sensitivities and specificities of the RDTs in this study were not statistically different depending on whether cultures were performed with or without APW, except for the sensitivity of the SD Bioline RDT, which was paradoxically lower when specimens were APW-enriched. Although this result was statistically significant, samples sizes were small and variable: 23 SD Biolne tests compared to APW-enriched bacterial cultures opposed to 428 tests compared to APW non-enriched cultures.

This study presents performance characteristics for three RDTs for cholera, evaluated in a regional laboratory in Haiti. We found that the Artron RDT is highly sensitive with poor to moderate specificity and similar to the widely-used Crystal VC RDT, while SD Bioline RDT has poor sensitivity and high specificity. We also show that Crystal VC and Artron RDTs frequently give false positive O139 results. Our findings reinforce conclusions from the Global Task Force for Cholera Control that in their current form, RDTs for cholera are insufficiently accurate to be used as point-of-care diagnostic tests, and should be limited to outbreak detection, surveillance and research. However, knowledge of the performance characteristics of these tests can help improve clinical, epidemiological and research practices in support of cholera control efforts. For example, the frequently documented occurrence of O139 false-positive results should be stressed when training users of the tests. Alternatively, RDT dipsticks and cassettes that only test for O1 or O139 are available and should be considered for use in settings where epidemics are dominated by a single serogroup. Both measures would reduce errors in interpretation by field users. RDT evaluations have also demonstrated that the performance characteristics of these tests can be highly variable, as seen in the low sensitivity and high specificity of SD Bioline RDT, highlighting the importance of site-specific evaluation of these tests prior to their use. These immediate changes in implementation can have significant impact on the utility of RDTs. In the end however, the absence of a rapid, affordable, easy-to-use and accurate test for the diagnosis of cholera remains a significant gap in our compendium of tools to combat global cholera epidemics; research to develop improved diagnostics will continue to remain a priority in the field.

## Supporting information

S1 Dataset Manuscript dataset. (XLSX)

S2 Dataset Dataset legend. (DOCX)

S1 Table Positivity rates for bacterial culture and 3 RDTs for detection of *V*. *cholerae* O1. (DOCX)

S2 Table Number of specimens tested by different combinations of RDTs. (DOCX)

## References

[1] Ali M, Nelson AR, Lopez AL, Sack DA. Updated global burden of cholera in endemic countries. *PLoS Negl Trop Dis*. 2015;9(6):e0003832 doi: 10.1371/journal.pntd.0003832 26043000 10.1371/journal.pntd.0003832 PMC4455997

[2] Koski-Karell V, Farmer PE, Isaac B, Campa EM, Viaud L, Namphy PC, et al Haiti’s progress in achieving its 10-year plan to eliminate cholera: hidden sickness cannot be cured. *Risk Manag Healthc Policy*. 2016 5;9:87–100. doi: 10.2147/RMHP.S75919 27307774 10.2147/RMHP.S75919 PMC4888864

[3] World Health organization—WHO. Prevention and Control of Cholera Outbreaks: WHO Policy and Recommendations. 2013. Available from: http://www.who.int/cholera/technical/prevention/control/en/

[4] Alam M, Hasan NA, Sultana M, Nair GB, Sadique A, Faruque ASG, et al Diagnostic limitations to accurate diagnosis of cholera. *J Clin Microbiol*. 2010;48(11):3918–22. doi: 10.1128/JCM.00616-10 20739485 10.1128/JCM.00616-10 PMC3020846

[5] Keddy KH, Sooka A, Parsons MB, Njanpop-Lafourcade BM, Fitchet K, Smith AM. Diagnosis of V*ibrio cholerae* O1 infection in Africa. *J Infect Dis*. 2013;208(SUPPL. 1):23–31. 10.1093/infdis/jit196 24101641

[6] Page A-L, Alberti KP, Mondonge V, Rauzier J, Quilici M-L, Guerin PJ. Evaluation of a Rapid Test for the Diagnosis of Cholera in the Absence of a Gold Standard. *PLoS One*. 2012;7(5):e37360 doi: 10.1371/journal.pone.0037360 22666350 10.1371/journal.pone.0037360 PMC3364251

[7] World Health Organization—The Global Task Force for Cholera Control. Interim Technical Note—The Use of Cholera Rapid Diagnostic Tests. 2016 p. 1–5.

[8] Dick MH, Guillerm M, Moussy F, Chaignat C-L. Review of two decades of cholera diagnostics—how far have we really come? *PLoS Negl Trop Dis*. 2012;6(10):e1845 doi: 10.1371/journal.pntd.0001845 23071851 10.1371/journal.pntd.0001845 PMC3469466

[9] Kalluri P, Naheed A, Rahman S, Ansaruzzaman M, Faruque ASG, Bird M, et al Evaluation of three rapid diagnostic tests for cholera: Does the skill level of the technician matter? *Trop Med Int Heal*. 2006;11(1):49–55. 10.1111/j.1365-3156.2005.01539.x 16398755

[10] Harris JR, Cavallaro EC, De Nóbrega AA, Jean JCB, Bopp C, Parsons MB, et al Field evaluation of crystal VC® rapid dipstick test for cholera during a cholera outbreak in Guinea-Bissau. *Trop Med Int Heal*. 2009;14(9):1117–21. 10.1111/j.1365-3156.2009.02335.x 19624473

[11] Sinha A, Sengupta S, Ghosh S, Basu S, Sur D, Kanungo S, et al Evaluation of a rapid dipstick test for identifying cholera cases during the outbreak. *Indian J Med Res*. 2012;135(4):523–8. 22664501 PMC3385237

[12] Boncy J, Rossignol E, Dahourou G, Hast M, Buteau J, Stanislas M, et al Performance and utility of a rapid diagnostic test for cholera: Notes from Haiti. *Diagn Microbiol Infect Dis*. 2013;76(4):521–3. doi: 10.1016/j.diagmicrobio.2013.03.010 23886437 10.1016/j.diagmicrobio.2013.03.010

[13] Mukherjee P, Ghosh S, Ramamurthy T, Bhattacharya MK, Nandy RK, Takeda Y, et al Evaluation of a rapid immunochromatographic dipstick kit for diagnosis of cholera emphasizes its outbreak utility. *Jpn J Infect Dis*. 2010;63(4):234–8. 20657061

[14] Ley B, Khatib AM, Thriemer K, von Seidlein L, Deen J, Mukhopadyay A, et al Evaluation of a rapid dipstick (Crystal VC) for the diagnosis of Cholera in Zanzibar and a comparison with previous studies. *PLoS One*. 2012;7(5):3–10. 10.1371/journal.pone.0036930 PMC3360732 22662131

[15] Hasan JA, Huq A, Nair GB, Garg S, Mukhopadhyay a. K, Loomis L, et al Development and testing of monoclonal antibody-based rapid immunodiagnostic test kits for direct detection of *Vibrio cholerae* O139 synonym Bengal. *J Clin Microbiol*. 1995 11;33(11):2935–9. 8576349 10.1128/jcm.33.11.2935-2939.1995 PMC228610

[16] Qadri F, Hasan JA, Hossain J, Chowdhury A, Begum YA, Azim T, et al Evaluation of the monoclonal antibody-based kit Bengal SMART for rapid detection of *Vibrio cholerae* O139 synonym Bengal in stool samples. *J Clin Microbiol*. 1995 3;33(3):732–4. 7751386 10.1128/jcm.33.3.732-734.1995 PMC228023

[17] Hasan JA, Huq A, Tamplin ML, Siebeling RJ, Colwell RR. A novel kit for rapid detection of *Vibrio cholerae* O1. *J Clin Microbiol*. 1994 1;32(1):249–52. 8126193 10.1128/jcm.32.1.249-252.1994 PMC263010

[18] Steenland MW, Joseph G a., Lucien MAB, Freeman N, Hast M, Nygren BL, et al Laboratory-confirmed cholera and rotavirus among patients with acute diarrhea in four hospitals in Haiti, 2012–2013. *Am J Trop Med Hyg*. 2013;89(4):641–6. doi: 10.4269/ajtmh.13-0307 24106190 10.4269/ajtmh.13-0307 PMC3795093

[19] Alam MT, Ray SS, Chun CN, Chowdhury ZG, Rashid MH, Madsen Beau De Rochars VE, et al Major Shift of Toxigenic *V*. *cholerae* O1 from Ogawa to Inaba Serotype Isolated from Clinical and Environmental Samples in Haiti. *PLoS Negl Trop Dis*. 2016 10;10(10):e0005045 doi: 10.1371/journal.pntd.0005045 27716803 10.1371/journal.pntd.0005045 PMC5055329

[20] Lesmana M, Richie E, Subekti D, Simanjuntak C, Walz SE. Comparison of direct-plating and enrichment methods for isolation of *Vibrio cholerae* from diarrhea patients. *J Clin Microbiol*. 1997 7;35(7):1856–8. 9196208 10.1128/jcm.35.7.1856-1858.1997 PMC229856

[21] Chen W, Zhang J, Lu G, Yuan Z, Wu Q, Li J, et al Development of an immunochromatographic lateral flow device for rapid diagnosis of *Vibrio cholerae* O1 serotype Ogawa. *Clin Biochem*. 2014;47(6):448–54. doi: 10.1016/j.clinbiochem.2013.12.022 24389075 10.1016/j.clinbiochem.2013.12.022

[22] George CM, Rashid M, Sack DA, Sack RB, Saif-Ur-Rahman KM, Azman AS, et al Evaluation of enrichment method for the detection of *Vibrio cholerae* O1 using a rapid dipstick test in Bangladesh. *Trop Med Int Health*. 2014 3;19(3):301–7. doi: 10.1111/tmi.12252 24401137 10.1111/tmi.12252 PMC4065385

[23] Debes AK, Ateudjieu J, Guenou E, Ebile W, Sonkoua IT, Njimbia AC, et al Clinical and Environmental Surveillance for *Vibrio cholerae* in Resource Constrained Areas: Application During a 1-Year Surveillance in the Far North Region of Cameroon. *Am J Trop Med Hyg*. 2016 3;94(3):537–43. doi: 10.4269/ajtmh.15-0496 26755564 10.4269/ajtmh.15-0496 PMC4775888

[24] Ontweka LN, Deng LO, Rauzier J, Debes AK, Tadesse F, Parker LA, et al Cholera Rapid Test with Enrichment Step Has Diagnostic Performance Equivalent to Culture. *PLoS One*. 2016;11(12):e0168257 doi: 10.1371/journal.pone.0168257 27992488 10.1371/journal.pone.0168257 PMC5167375

[25] Ansaruzzaman M, Shimada T, Bhuiyan N a., Nahar S, Alam K, Islam MS, et al Cross-reaction between a strain of *Vibrio mimicus* and *V*. *cholerae* O139 Bengal. *J Med Microbiol*. 1999 9;48(9):873–7. doi: 10.1099/00222615-48-9-873 10482300 10.1099/00222615-48-9-873

[26] Albert MJ, Ansaruzzaman M, Shimada T, Rahman A, Bhuiyan NA, Nahar S, et al Characterization of *Aeromonas trota* strains that cross-react with *Vibrio cholerae* O139 Bengal. *J Clin Microbiol*. 1995 12;33(12):3119–23. 8586685 10.1128/jcm.33.12.3119-3123.1995 PMC228656

