# Supplementary material for: Laboratory evaluation of immunochromatographic rapid diagnostic tests for cholera in Haiti
Source: PLoS One. 2017 Nov 1;12(11):e0186710. doi: 10.1371/journal.pone.0186710 (PMC5665506; doi:10.1371/journal.pone.0186710)
Supplement: S2 Dataset — (DOCX) [file pone.0186710.s002.docx]

**Dataset Legend**

*Culture*

1. ogawa = Ogawa. These bacterial cultures were positive for *V. cholerae* serotype Ogawa.
2. inaba = Inaba. These bacterial cultures were positive for *V. cholerae* serotype Inaba.
3. prob = Probable. These bacterial cultures grew specimens that were isolated on TCBS, HIA, and had a positive oxidase and string test, but were negative on serologic testing with antisera for *V. cholerae* O1 and O139.
4. neg = Negative. Cultures showed no growth.
5. np = Not performed. Bacterial culture was not performed.
6. inv = Invalid. Documented bacterial culture results were incomplete and/or illegible.

*Crystal VC, SDBioline, Artron*

1. o1 = positive band on the rapid test section corresponding to *V. cholerae* serogroup O1.
2. o139 = positive band on the rapid test section corresponding to *V. cholerae* serogroup O139.
3. o1o139 = positive bands on the rapid test sections corresponding to *V. cholerae* serogroups O1 and O139.
4. neg = Negative. Absence of positive bands, apart from the control band.
5. np = Not performed. RDT was not performed for this specimen.
6. inv = Invalid. Specimens excluded from analysis. For Crystal VC, invalid results were excluded from the analysis because results were incompletely documented. For SD Bioline, results were excluded from the analysis because tests were performed past their expiration date.

APW

1. APW = alkaline peptone water.
2. 0 = APW enrichment was not performed prior to culture.
3. 1 = APW enrichment was performed prior to culture.
