# Supplementary material for: Laboratory evaluation of immunochromatographic rapid diagnostic tests for cholera in Haiti
Source: PLoS One. 2017 Nov 1;12(11):e0186710. doi: 10.1371/journal.pone.0186710 (PMC5665506; doi:10.1371/journal.pone.0186710)
Supplement: S1 Table — (DOCX) [file pone.0186710.s003.docx]

**Supporting Table 1****. Positivity Rates for Bacterial Culture and 3 RDTs for detection of *V. cholerae* O1.**

|  | Without APW enrichment | | | | With APW enrichment | | | | Grouped | | | |
| --- | --- | --- | --- | --- | --- | --- | --- | --- | --- | --- | --- | --- |
| Test | Total | Positive | Negative | Positivity Rate (%) | Total | Positive | Negative | Positivity Rate (%) | Total | Positive | Negative | Positivity Rate (%) |
| Culture | 450 | 234 | 216 | 52.0 | 455 | 243 | 212 | 53.4 | 905 | 477 | 428 | 52.7 |
| VC | 349 | 239 | 110 | 68.5 | 162 | 108 | 54 | 66.7 | 511 | 347 | 164 | 67.9 |
| Art | 9 | 5 | 4 | 55.6 | 120 | 85 | 35 | 70.8 | 129 | 90 | 39 | 69.8 |
| SD | 428 | 204 | 224 | 47.7 | 23 | 8 | 15 | 34.8 | 451 | 212 | 239 | 47.0 |
