# Supplementary material for: Laboratory evaluation of immunochromatographic rapid diagnostic tests for cholera in Haiti
Source: PLoS One. 2017 Nov 1;12(11):e0186710. doi: 10.1371/journal.pone.0186710 (PMC5665506; doi:10.1371/journal.pone.0186710)
Supplement: S2 Table — (DOCX) [file pone.0186710.s004.docx]

**Supporting Table 2. Number of Specimens tested by different combinations of RDTs.**

| VC | VC + SD | VC + Art | VC + SD + Art | SD | SD + Art | Art |
| --- | --- | --- | --- | --- | --- | --- |
| 19 | 363 | 120 | 9 | 79 | 0 | 0 |

*VC = Crystal VC; SD = SD Bioline; Art = Artron
